# Resistance to Vip3Aa: A Growing Threat with Unclear Mechanisms and Management Implications

**DOI:** 10.3390/insects16080820

**Published:** 2025-08-07

**Authors:** Rajeev Roy, Dawson Kerns, Juan Luis Jurat-Fuentes

**Affiliations:** Department of Entomology and Plant Pathology, University of Tennessee, 370 Plant Biotechnology Building, 2505 E. J. Chapman Drive, Knoxville, TN 37996, USA; rajeev_roy@imbb.forth.gr (R.R.); ddkerns@agcenter.lsu.edu (D.K.)

**Keywords:** Vip3Aa, resistance, *Bacillus thuringiensis*, vegetative insecticidal protein

## Abstract

The vegetative insecticidal protein Vip3Aa from *Bacillus thuringiensis* (Bt) has become a key plant-incorporated protectant (PIP) in transgenic crops targeting lepidopteran pests, particularly as resistance increasingly compromises the efficacy of Cry protein PIPs. More than a decade after its commercial deployment, Vip3Aa performance remains efficacious but increasingly vulnerable. Field screens have detected unexpectedly high baseline frequencies of Vip3Aa-resistant alleles and have produced highly resistant strains in several major pests, including *Helicoverpa* spp., *Spodoptera* spp., and *Mythimna separata*. Although structure–function experiments and studies on resistance to Vip3Aa have identified altered midgut processing and impaired receptor binding as candidate resistance mechanisms, the underlying genetic determinants remain poorly understood. Moreover, resistance to Vip3Aa appears to diverge from canonical Cry protein resistance pathways, underscoring the need for dedicated mechanistic studies. This review critically examines the available experimental evidence on Vip3Aa resistance mechanisms, highlighting major knowledge gaps and proposing research priorities to inform resistance monitoring and extend the durability of Vip3Aa-based pest control.

## Simple Summary

Insect-resistant transgenic crops expressing Cry and Vip3Aa insecticidal proteins from the bacterium *Bacillus thuringiensis* help farmers protect yields from pests while reducing reliance on chemical pesticides. The Vip3Aa protein has been especially useful against caterpillar pests that damage crops like corn and cotton, especially after documented cases of resistance to Cry proteins in these pests. However, there are growing concerns because some insects are starting to survive exposure to Vip3Aa, underscoring the risk of resistance emergence and the urgent need to understand the mechanisms, inheritance, and spread of resistance. A critical review of available information on Vip3Aa resistance is timely and essential, as it informs resistance management strategies and guides future research efforts in insect resistance management. The goal of this review is to provide a clear understanding of what is known so far about resistance to Vip3Aa and what gaps remain in our knowledge.

## 1. Introduction

The entomopathogenic bacterium *Bacillus thuringiensis* (Bt) produces diverse insecticidal proteins that are classified based on both amino acid sequence identity and structural similarity [1]. Most known Bt insecticidal proteins belong to the Cry toxin family and are produced during the sporulation phase of the bacterium and stored in parasporal crystals, while members of the vegetative insecticidal protein (Vip) family are produced and secreted during the vegetative phase of growth [2]. Sprayable mixtures of Bt spores and proteins have been used for pest control for more than 80 years, but the largest impact of Bt proteins in agriculture is through their expression in transgenic plants as plant-incorporated protectants (PIPs). Transgenic plants expressing Cry and Vip3Aa insecticidal protein genes, generically called “Bt crops”, are planted in more than 101 million hectares globally [3] for effective pest control, increased yields, and reduced mycotoxin contamination [4,5,6]. Since Cry and Vip3Aa PIPs have distinct modes of action [7], they are ideal candidates for co-expression through gene pyramiding to hinder the evolution of resistance [2,8], currently the most serious threat to the sustainability of Bt crops.

Field failures have already been documented for selected Cry PIPs in some locations [9], increasing reliance and, as a consequence, selection pressure for the evolution of resistance to Vip3Aa. For instance, practical resistance to Cry1A PIPs in populations of the corn earworm (*Helicoverpa zea*) in the U.S. [10,11] makes Vip3A the only remaining PIP effective against this pest in some locations [12]. While there are no published reports of field-evolved practical resistance in the U.S., there are some early indications of reduced efficacy of Vip3Aa PIPs against *H. zea* [13]. Similarly, populations of the fall armyworm (*Spodoptera frugiperda*) have evolved practical resistance to multiple Cry PIPs [9], and unofficial reports of reduced efficacy of Vip3Aa PIPs are emerging in Brazil [14]. These reports suggest that field resistance to Vip3Aa PIPs may be looming, at a time when information on resistance genes and mechanisms that may lead to field failures is scarce.

**Figure 1:**
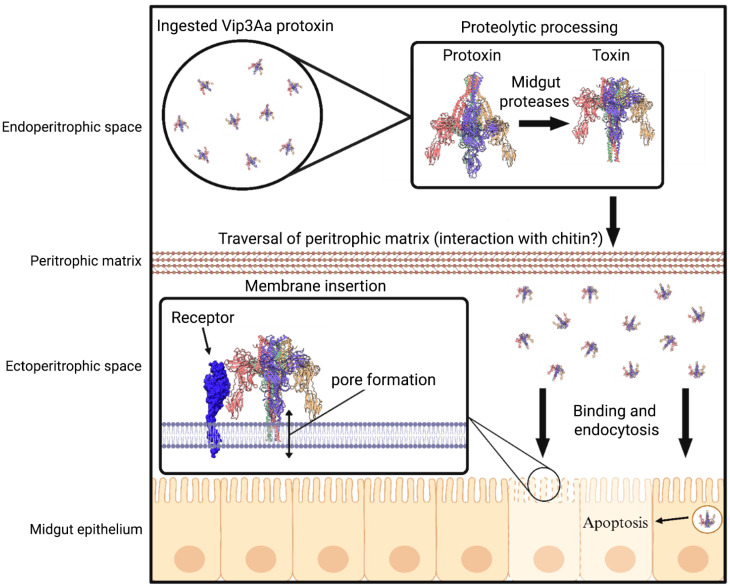
Diagram of the Vip3Aa mode of action. The Vip3Aa protoxin is produced by *B. thuringiensis* as a soluble tetramer, which after ingestion by a susceptible insect undergoes processing by gut proteases. This process triggers the unfurling of a needle-like projection in the toxin structure. The Vip3Aa toxin must then interact with chitin and traverse the peritrophic matrix to reach receptors on the surface of midgut cells. The needle-like projection of the toxin then inserts into the cell membrane forming a pore that leads to cell lysis by osmotic shock. There is evidence from insect cell cultures that Vip3Aa toxin is endocytosed and induces cell death by apoptosis. The Vip3Aa protoxin (entry: 6TFJ) and activated toxin (entry: 6TFK) structures shown were obtained from the Worldwide Protein Data Bank (https://www.wwpdb.org/). Distinct structural domains in the Vip3Aa protoxin and toxin structures are shown with different colors.

In principle, any alteration in the multi-step mode of action of Vip3Aa could potentially result in resistance. The most widely accepted model (Figure 1) includes proteolytic processing, interactions with chitin and receptors on the midgut brush border membrane, insertion into the cell membrane to form a pore, and cell death by osmotic shock [15].

Both Cry and Vip3Aa proteins are pore-forming toxins, yet they recognize distinct midgut binding sites [16], explaining their lack of cross-resistance [17]. In this review, we critically examine the available evidence on resistance to the Vip3Aa toxin, organized according to the sequential steps in its mode of action.

## 2. Altered Protoxin Processing

Upon ingestion, the Vip3Aa protoxin requires processing by serine proteases in the midgut fluids of the host, although this step is sufficient for toxicity, as the protoxin is also processed by gut fluids from non-susceptible insects [18]. The protoxin form (~89 kDa) forms a pyramid-shaped tetramer in solution that includes five distinct domains (I to V), with Domains I and II having α-helical structures and domains III-V being mostly composed of β-sheets [19].Processing by cationic chymotrypsin and anionic trypsin in the host gut fluids [20] renders a toxin form consisting of two fragments that remain associated: a ~22 kDa fragment from domain I, and the rest of the protein (~66 kDa). This processing also results in a conformational shift of the protoxin into the toxin tetramer, with all four domains I forming an extended needle structure expected to insert into the target cell membrane [19].

Initial evidence suggested that processing of Vip3Aa protoxin to toxin is necessary for insecticidal activity [21], and that differences in processing efficacy may help explain distinct susceptibility to Vip3Aa among closely related species [22]. However, several studies with cultured Sf9 cells detected Vip3Aa protoxin internalization associated with apoptosis, suggesting that processing was not needed for cytotoxicity [23,24,25,26]. In contrast, more recent results from studies using Sf9 cells and midgut brush border membrane vesicle (BBMV) proteins from *Spodoptera* spp. demonstrate that while both Vip3Aa protoxin and toxin can interact with the BBMV and cultured Sf9 cells, protoxin binding is mostly non-specific and does not result in Sf9 cell lysis [27]. This discrepancy in the activity of Vip3Aa protoxin may be explained by the presence of small amounts of contaminating Vip3Aa toxin within the protoxin samples tested. In fact, the purification of pure Bt protoxins is difficult, as the bacterium produces endogenous serine proteases to initiate processing before exposure to host gut fluids. In most of these studies [23,24,25], protease inhibitors were not used during Vip3Aa purification and, consequently, there is slight active toxin contamination present that may account for the difference in activity. However, Nimsanoor et al. [26] explicitly added a protease inhibitor in their purification so that no activated toxin was present, and their results are probably indicative of protoxin activity.

**Figure 2:**
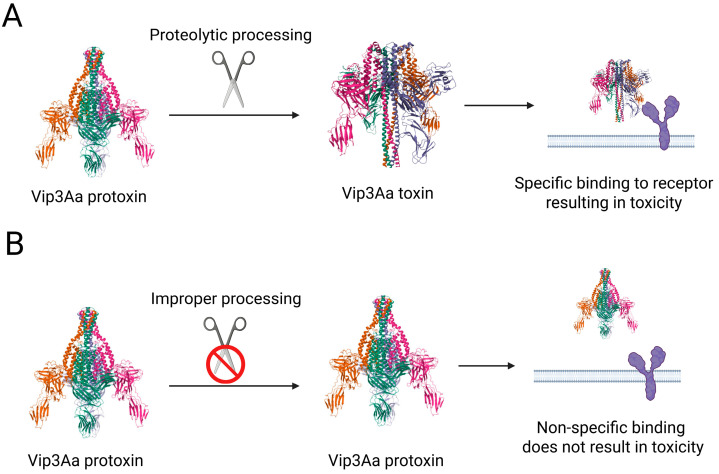
Conceptual model illustrating resistance due to lack of Vip3Aa protoxin processing. The processing of Vip3Aa protoxin in susceptible insects (**A**) is interrupted in resistant insects (**B**), resulting in a lack of effective binding to receptors (i.e., conducive to insertion) on the midgut membrane. Distinct structural domains in the Vip3Aa protoxin and toxin structures are shown with different colors.

While still a relatively low number of cases have been studied, slower protoxin processing relative to susceptible individuals (Figure 2) appears common in Vip3Aa-resistant larvae, with examples reported in strains of *Spodoptera litura* [28], *Helicoverpa armigera* [29], *Chloridea* (*Heliothis*) *virescens* [30], and *S. frugiperda* [31]. Direct evidence supporting the impact of reduced protoxin processing in resistance has been presented in *S. frugiperda*, where pre-processing of Vip3Aa protoxin with midgut fluids from susceptible insects significantly increased toxicity against resistant larvae [31]. This observation agrees with Vip3Aa protoxin being inactive and requiring processing to the active toxin for effective binding and toxicity [32]. One potential approach to overcome this mechanism is engineering the Vip3Aa protein to undergo formation of the toxin structure in solution without depending on processing by serine proteases. Such a mutant was developed by removing alpha helix 1 from Domain I of Vip3Aa [33], which bound to Vip3Aa receptors and had increased irreversible binding compared to the wild-type toxin [27]. However, this mutant had >16-fold lower activity against *Spodoptera exigua* larvae, underscoring the importance of helix 1 in Vip3Aa and supporting that the toxin structure is needed but not sufficient for toxicity. Another Vip3Aa protein variant carrying the M34L mutation inside helix α1 showed enhanced toxicity in *S. exigua* [33], but its structure in solution was not reported. Mutant Vip3Aa protoxins containing additional trypsin cleavage sites between domains I and II had faster processing and increased toxicity against *S. frugiperda* and *H. armigera* [34], but the activity of these mutants against Vip3Aa-resistant strains with altered processing has not been reported.

Slower Vip3Aa processing in midgut fluids from resistant compared to susceptible larvae is associated with reduced expression of specific protease genes and total serine protease activity. Interestingly, reduced serine protease activity in Vip3Aa-resistant *S. frugiperda* did not affect the processing of Cry1F, supporting that distinct protease genes are involved in Vip3Aa and Cry processing. Supporting this hypothesis, resistance to Cry1C and Vip3Aa in *S. litura* was associated with reduced activity of distinct protease bands in zymograms [28]. Additionally, there was no cross-resistance to Cry proteins in larvae resistant to Vip3Aa with reduced protease activity and Vip3Aa processing [8]. Similarly, negligible cross-resistance between Vip3Aa and Cry toxins has been detected in other Bt-resistant larvae [17,35].

Evidence from transcriptomic analyses by HT-SuperSAGE in *C. virescens* [30] and RNA-Seq in *S. frugiperda* [31] also supports highly reduced expression of specific serine protease genes in Vip3Aa-resistant larvae, with a concomitant over-expression of alternative serine protease genes. This apparent compensatory response has been previously documented among lepidopteran larvae [36,37]. While this response may not affect Vip3Aa toxicity, it may impact the processing and activity of other insecticidal proteins. For instance, Vip3Aa-resistant *S. frugiperda* with altered Vip3Aa processing and reduced expression of serine protease genes [31] had increased susceptibility to Cry1F and Cry2Ae [8]. While not broadly tested, this negative cross-resistance may be contributing to mask resistance to Vip3Aa and extend the durability of current pyramided Vip3Aa and Cry traits in Bt crops.

## 3. Interactions with the Peritrophic Matrix

The Vip3Aa toxin interacts with chitin on the peritrophic matrix through domain V, and this uncharacterized interaction appears critical for toxicity [38]. Thus, a truncated protein containing Domains I to III of the Vip3Aa toxin is cytotoxic but inactive in vivo [38]. In agreement with the importance of chitin-Vip3Aa interactions for toxicity, a retrotransposon-mediated insertion in the chitin synthase-2 (CHS-2) gene resulted in reduced peritrophic matrix formation and very high levels of resistance to Vip3Aa in *S. frugiperda* [39]. Genetic knockout of CHS-2 by CRISPR/Cas9 gene editing resulted in a more complete absence of the peritrophic matrix and a similar resistance phenotype [40]. This observation has been replicated in other lepidopteran insect pest models [41]. Since CHS-2 does not serve as a Vip3Aa receptor when expressed in a heterologous system [42], the high levels of resistance from the altered CHS-2 are most probably related to the resulting reduced chitin presence.

While knocking out CHS-2 results in high levels of resistance to Vip3Aa in all insects evaluated, it also incurs relevant fitness costs that would prevent this mutation from being naturally selected in agricultural settings [41]. Alleles affecting the expression of CHS-2 have already been detected in field *S. frugiperda* samples from China; the resulting phenotype differed from a CHS-2 gene knockout and presented a reduced but not completely absent peritrophic matrix [39]. In contrast, CHS-2 expression or midgut chitin content was unaltered in Vip3a-resistant *S. frugiperda* from the U.S.A. [31]. Lack of involvement of CHS-2 alterations in Vip3Aa resistance is also supported by the lack of relevant fitness costs in resistant *S. frugiperda* from the U.S.A. [43]. Targeted DNA-based monitoring [44] of this gene [39] to detect potential mutations leading to either downregulation or loss of function would provide more information on the relevance of CHS-2 alleles in field settings.

## 4. Binding to Receptors in the Midgut

**Figure 3:**
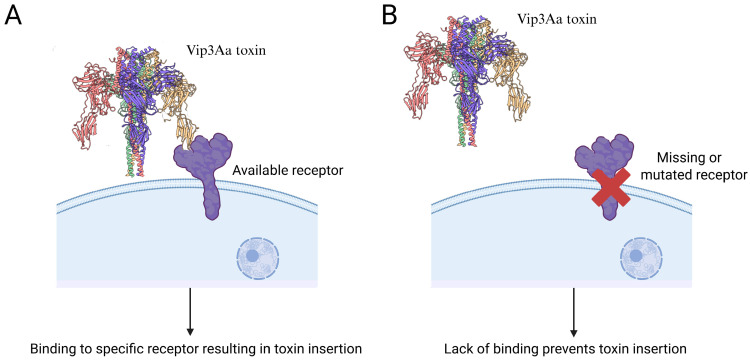
A diagram describing resistance to Vip3Aa due to a lack of toxin binding. The Vip3Aa toxin must bind to receptor(s) on the midgut cell membrane to form pores and cause cell lysis by osmotic shock (**A**). In the resistant insect (**B**), the specific Vip3Aa receptor is either missing, mutated, or downregulated, resulting in reduced productive Vip3Aa binding (i.e., resulting in toxin insertion). Distinct structural domains in the Vip3Aa toxin structure are shown with different colors.

The Vip3Aa toxin recognizes specific receptors on the insect midgut epithelium as a step required for subsequent pore formation [15], but not sufficient for toxicity, as binding was also detected in Vip3Aa-resistant strains of some species [29,45]. Commonly, binding is tested using in vitro binding experiments with labeled Vip3Aa toxin and midgut brush border membrane vesicles (BBMVs) from lepidopteran larvae [22,46,47,48]. Reduced binding to receptors in these tests is the most common mechanism of resistance observed for Cry toxins [49] and can potentially be an in vitro indication of resistance to Vip3Aa (Figure 3).

Reduced, but not absent, binding has only been experimentally observed in Vip3Aa-resistant strains of *H. zea* and *S. frugiperda* [31,50], although the specific mechanism or receptor(s) involved are not known. To date, no receptor protein has been functionally validated in vivo as a Vip3Aa receptor. Experiments performed in cultured Sf9 cells identified multiple candidate receptors, including the ribosomal S2 (RBS-2) protein [51], scavenger receptor-c (SRC) [24], prohibitin-2 (PHB-2) [52], fibroblast growth factor receptor (FGFR) [53], and tenascin-like glycoprotein [54,55]. However, this identification of putative Vip3Aa receptors from cultured cells (i.e., Sf9) needs to be taken with caution, as so far, they have not extended to in vivo conditions. For instance, *S. frugiperda* larvae with genetic knockouts of FGFR or SRC had unaltered susceptibility to Vip3Aa compared to reference susceptible strains [56]. Further research is needed to identify functional Vip3Aa receptors, which will advance the characterization of resistance due to binding alterations.

## 5. Mechanism of Cytotoxicity

There are presently two proposed post-binding mechanisms of Vip3Aa toxicity. The predominant model suggests that Vip3Aa toxin forms pores on the midgut epithelial membrane that cause cell death by osmotic shock [57,58,59]. The pore formation ability of Vip3Aa has been experimentally established in both in vitro and in vivo models [32,57,58]. Alternatively, studies with cultured cell line models detected Vip3Aa internalization and apoptosis induction [23,24,25,26]. Apoptosis was also detected after treatment with a sublethal dosage of Vip3Aa in 4th instar *S. exigua*, but this was not observed with higher Vip3Aa concentrations [60]. One possible explanation for these disparate observations is that apoptosis may not be responsible for in vivo toxicity but instead represent a defensive response to eliminate Vip3Aa-affected midgut cells, as observed with Cry toxins [61]. Endocytosis of Vip3Aa in cultured cells was shown to involve the SRC and FGFR proteins [24,53], while interactions with RBS-2 seem critical for cytotoxicity [51].

An integrative explanation is that apoptosis and pore formation are not mutually exclusive and that their impact on toxicity may depend on Vip3Aa concentration. Lower relative Vip3Aa concentrations bound to receptors on the cell membrane may proceed to endocytosis, which then may be followed by apoptosis. In contrast, higher Vip3Aa concentrations would facilitate pore formation and disruption of the cell membrane, hindering endocytosis. A similar integrative process was proposed for the pore formation and oncotic mechanisms in the Cry intoxication process [62]. Further research is needed to understand if both Vip3Aa pore formation and apoptotic mechanisms function simultaneously, their relative contributions to toxicity, and to identify the specific pathways involved in Vip3Aa-induced apoptosis.

## 6. Other Critical Genes Involved in Resistance to Vip3Aa

Recently, a gene that is critical to Vip3Aa susceptibility in *H. armigera* has been identified, encoding an uncharacterized protein (HaVipR1) [63]. This gene is not expressed in Sf9 cells, and its specific role in the Vip3Aa intoxication process remains unknown. Phylogenetic analysis indicates that HaVipR1 homologs are relatively conserved within Lepidoptera but distinct from other insects [63]. Knockout of the homologous gene in *S. frugiperda* also causes Vip3Aa resistance [64], experimentally supporting that VipR1 may be a common Vip3Aa resistance gene in Lepidoptera. Lepidopteran members of the VipR1 family contain two thyroglobulin domains and a calcium-binding domain. While the specific role of HaVipR1 in resistance to Vip3Aa is so far unknown, the protein is predicted to be targeted to the extracellular space [63,65], suggesting that it may impact pre-binding steps in the mode of action. This is supported by the lack of Vip3Aa binding differences when comparing a strain of *H. armigera* with a disrupted HaVipR1 gene with a susceptible population [29]. Further research is needed to mechanistically describe resistance to Vip3Aa due to VipR1 disruption.

While membrane-bound alkaline phosphatase (ALP) in the midgut of lepidopteran insects does not bind Vip3Aa [54,55,66], a reduction in ALP expression was associated with resistance to Vip3Aa in *C. virescens* [66]. While the role of reduced ALP expression in resistance to Vip3Aa remains unclear, a similar phenotype has been described in Cry-resistant lepidopteran larvae [67,68,69,70]. However, this reduced ALP expression was not linked with resistance to Cry1F in *S. frugiperda* [71], and ALP expressed in cultured insect cells did not serve as a Vip3Aa receptor [66]. These observations suggest that reduced ALP levels may represent a compensatory mutation unrelated to the resistance mechanism.

Reduced expression of the myeloblastosis transcription factor gene (*SfMyb*) was associated with resistance to Vip3Aa in a strain of *S. frugiperda* [72]. Among other genes, *SfMyb* impacts the expression of RBS-2 [51,72], which, according to data from cultured insect cells, can affect susceptibility to Vip3Aa [51]. Altered expression of serine protease genes regulated by *SfMyb* could also be involved in reduced processing and resistance to Vip3Aa in *S. frugiperda* [31]. Other genes that may be regulated by *SfMyb* and play a functional role in resistance to Vip3Aa need to be identified.

## 7. Current Status of Field Resistance to Vip3Aa

Even before its commercial use, Vip3Aa resistance allele frequency in field populations of *H. armigera* and *Heliothis punctigera* in Australia was higher than the initial optimal frequency (<0.00001) assumed for the high-dose/refuge resistance management strategy used for Bt crops [73]. This observation underscored an elevated risk for resistance evolution and the need for effective management approaches. Moreover, similar observations have been reported for other locations and insect pests. Between 2015 and 2017, the Vip3Aa resistance allele frequency for *S. frugiperda* in Brazil increased from 0.0009 [74] to 0.0033 [75]. Estimates of the Vip3Aa resistance allele frequency for the same species in the U.S. ranged between 0.0048 and 0.0072 [76]. Estimates of resistance allele frequency for *H. zea* from F2 screens reach 0.0155 [77], and even higher estimates (0.115 to 0.032, depending on location) are observed in sentinel corn plots [2]. However, despite this higher-than-desirable frequency of resistance alleles, Vip3Aa events in Brazil [78] and the U.S. [2] remain effective in controlling target pest populations. This inconsistency may be explained by the relevant fitness costs associated with resistance to Vip3Aa under field conditions, as detected under laboratory conditions [79,80]. In addition, negative cross-resistance between Vip3Aa and Cry toxins may hinder the evolution of resistance to Vip3Aa in pyramided events [8].

Practical field-evolved resistance to Vip3Aa has yet to be reported, with available information on resistance deriving from laboratory selection of field-collected insects (Table 1). In most cases, resistance to Vip3Aa is monogenic and recessive, although polygenic resistance was described in strains of *C. virescens* [81], *H. zea* [79], and *M. separata* [82]. While monogenic resistance is autosomal, transmission of polygenic resistance to Vip3Aa has been shown to have maternal [82] and paternal [81] influences.

One of the more puzzling aspects of Vip3Aa resistance is the lack of shared mechanisms or causative genes, even when considering the same pest species and strains derived from neighboring geographic areas. For instance, Vip3Aa-resistant strains of *S. frugiperda* from Louisiana and Texas had resistance linked to different loci [83]. Similarly, distinct Vip3Aa resistance loci were identified in two *S. frugiperda* strains generated from insects collected in the same Chinese province [39,72]. In *H. zea*, three different loci were involved in resistance to Vip3Aa in five different strains generated from F2 screens of populations from Texas, Louisiana, and Mississippi [84]. One locus was shared between two strains from Stoneville (MS), and a second locus was unique to a strain from Winnsboro (LA). On the other hand, a third distinct resistance locus was shared by strains collected in Snook (TX) and Alexandria (LA) in the same year, suggesting dispersal of resistance between some *H. zea* populations. In 2021 and 2022, two separate resistant strains of *M. separata* were selected from insects collected in Jilin Province (China). While the specific locus involved in resistance in each strain is unknown, the strains showed distinct resistance evolution dynamics and ratios [45,82]. Taken together, reports of distinct resistance loci in closely located populations support the existence of multiple resistance loci in field populations. This diversity in resistance genes would be expected from a complex mode of action involving multiple critical steps, as seems to be the case for Vip3Aa [15]. However, this does not occur with Cry toxins, where reduced binding to ABC transporters or cadherins that serve as receptors is commonly found in cases of practical resistance [85]. Detailed mechanistic information and the identity of Vip3Aa receptors are needed to better explain the unusually high diversity in Vip3Aa resistance loci. Based on currently available experimental evidence from resistant strains, processing of Vip3Aa protoxin to toxin [31], and interactions with midgut chitin [39,41] and unidentified membrane receptors [50], appear critical steps for Vip3Aa intoxication. The identification of the specific genes involved in these steps will advance the development DNA-based monitoring for Vip3Aa resistance.

**Table 1:** Characteristics of Vip3Aa resistance in strains of different lepidopteran species. RR = resistance ratio estimated as the lethal concentration killing 50% of larvae (LC_50_) in resistant to susceptible strains. CRR = cross-resistance ratio estimated as for RR for a toxin not used during selection.

Species	Origin	RR	Inheritance	CRR *	Fitness Cost	Refs
*Chloridea virescens*	Field Mississippi 2006	2040	Paternal influence Polygenic	6.7 Cry1Ab 1.0 Cry1Ac	Reduced survival to adult, egg viability, and mating success	[81,86]
*Helicoverpa armigera*	F2 screen Australia 2009–2010	>232	Maternal influence Recessive Monogenic	1.7 Cry1Ac 0.3 Cry2Ab	NA	[29,65,73]
*Helicoverpa punctigera*	F2 screen Australia 2009–2010	>215	Autosomal Recessive Monogenic	3.2 Cry1Ac 1.7 Cry2Ab	NA	[73]
*Helicoverpa zea*	F2 screen Snook (TX) 2019	45,194	Autosomal Recessive Monogenic	0.2 Cry1A.105 1.6 Cry1Ac 0.5 Cry2Ab	NA	[87]
	F2 screen Alexandria (LA) 2019	>909	Autosomal Recessive Monogenic	NA	NA	[84]
	F2 screen Winnsboro (LA) 2020	>909	Autosomal Recessive Monogenic	NA	NA	[84]
	F2 screen Stoneville (MS) 2020	>909	Autosomal Recessive Monogenic	NA	NA	[84]
	Lab selection Georgia (USA) 2023	267	Autosomal Recessive	NA	Resistance reduced in heterogeneous strains without selection	[79]
*Spodoptera frugiperda*	F2 screen Brazil 2016	>3200	Autosomal Recessive Monogenic	Survives Cry1Ab + Vip3Aa20 corn	Reduced fecundity, survival, and reproductive rate	[74,80]
	F2 screen Rapides (LA) 2016	>632	Autosomal Recessive Monogenic	0.5 Cry1F 1.4 Cry2Ab 0.4 Cry2Ae	Reduced pupal weight and longer pupal development	[43,88]
	F2 screen Snook (TX) 2018	>395	Autosomal Recessive Monogenic	2.9 Cry2Ab 1.9 Cry1F	NA	[83,89]
	Field Tifton, (GA) 2012	>9800	NA	NA	Slower growth and lower pupation rate	[78]
	Lab selection Yunan (China) 2023	5562	Autosomal Recessive Monogenic	2.0 Cry1Ab 0.9 Cry1Ac 2.0 Cry1F 1.8 Cry2Ab	NA	[39]
	Lab selection Yunan (China) 2023	206	Autosomal Recessive	1.2 Cry1Ab 2.0 Cry1F 1.6 Cry2Ab	NA	[72]
*Mythimna separata*	Lab selection Jilin (China) 2021	>3061	NA	2.8 Cry1Ab 0.6 Cry1F	NA	[45]
	Lab selection Jilin (China) 2022	>634	Maternal influence Incomplete dominant Polygenic	NA	Longer larval development, lower pupation rate, adult emergence, and reproductive rate	[82]

* Values rounded to one decimal place from the original in publications.

## 8. Conclusions and Prospects

Widespread resistance to Cry proteins in major agricultural pests threatens the sustainability of Bt crops and their benefits [90], and heightens the pressure for the evolution of resistance to Vip3Aa traits. Negative cross-resistance with Cry proteins may be contributing to extending the field efficacy of Vip3Aa traits in pyramided Bt crops, although ongoing sentinel plot monitoring is detecting higher-than-expected plant damage and surviving larvae in some cases [2]. The increased risk of practical resistance to Vip3Aa underscores the urgency of identifying resistance genes to enhance monitoring and guide the design of more effective and sustainable Bt crops. However, currently available experimental evidence suggests disparity in resistance loci and mechanisms, even in the same pest species, indicating that Vip3Aa resistance is likely to be more diverse compared to resistance against Cry toxins [49].

Pyramided Bt crops producing multiple PIPs may select for resistance mechanisms that differ from laboratory selection with Vip3Aa alone. While receptor-related resistance mechanisms are typically expected for crops producing a single *Bt* protein [49], alterations in common steps in the mode of action of the pyramided PIPs would be expected to arise through selection with pyramided crops. Perhaps the most plausible mechanism affecting multiple PIPs would be an alteration of midgut proteases affecting protoxin processing. Since Vip3Aa resistance associated with altered processing does not affect susceptibility to Cry proteins [31], this mechanism is not plausible to evolve in pyramided crops. Other potential resistance mechanisms that could affect diverse Bt toxins could include toxin sequestration, increased excretion, increased detoxification, and behavioral changes. The emergence of resistance to pyramided PIPs would be more likely for regions where single-protein Bt crops were not widely used or where no resistance to single toxins was reported.

Another limitation in identifying field-relevant resistance mechanisms from laboratory studies is the dramatic difference in toxin concentration used for selection compared to the amount of toxin expressed in Bt crops. This is particularly true for traits considered as “low-dose” for a particular pest. For instance, Vip3Aa in Viptera MIR162 was described as a high-dose trait for *S. frugiperda,* and based on data in the registration package for this event, it contained approximately 180 μg of Vip3Aa per gram of leaf tissue [91].These Vip3Aa levels are much lower than the concentrations used in most studies to select for resistance in the laboratory [39,64,86,87,88], which contributes to selection of distinct mechanisms. Importantly, the Cry1Ab protein commonly pyramided with Vip3Aa exhibits low efficacy against *S. frugiperda*, suggesting that in this case a pyramid would effectively function as a single toxin event.

New proteins with a unique mode of action would provide ideal candidates for pyramiding with Vip3Aa to delay resistance in target lepidopteran pests. The eCry1Gb.1Ig chimeric protein shows efficacy against strains of *S. frugiperda* that are resistant to Cry1F, Cry1A.105, Cry2Ab2, and Vip3Aa [92] and represents an excellent candidate for pyramiding and extending the efficacy of Vip3Aa traits. Additionally, Cry1Da_7 and Cry1B.868 have been identified as ideal candidates for pyramiding due to their targeting of non-shared binding sites and toxicity against Vip3Aa- and Cry1F-resistant strains of *S. frugiperda* and *H. zea* [93].

Our knowledge of the Vip3Aa mode of action remains limited at a time when selection of resistance to Vip3Aa PIPs is ongoing. For example, interactions of Vip3Aa with midgut chitin appear critical for susceptibility, yet nothing is known about how or when this interaction occurs and, more importantly, why it dramatically impacts susceptibility. It could be that this interaction with chitin facilitates protoxin-to-toxin transition or protects the toxin from further processing. There are also no confirmed Vip3Aa midgut receptors, and the role of Vip3Aa-induced apoptosis in larval mortality remains unclear. Clearly, further research at the molecular level is needed to improve our understanding of the Vip3Aa mode of action and provide this information to improve resistance management. For instance, the identification of reliable resistance markers would allow for the deployment of highly sensitive and cost-effective DNA-based monitoring for more robust resistance management. Expanding our understanding of insect–Vip3Aa toxin interactions at the genomic and proteomic levels will not only enhance resistance mitigation efforts but also guide the development of next-generation PIPs.

## Data Availability

No new data were created.

## Abbreviations

The following abbreviations are used in this manuscript:

Bt	*Bacillus thuringiensis*
PIP	Plant incorporated protectant
BBMV	Brush border membrane vesicle

## References

[1] Crickmore N., Berry C., Panneerselvam S., Mishra R., Connor T.R., Bonning B.C. (2020). A structure-based nomenclature for *Bacillus thuringiensis* and other bacteria-derived pesticidal proteins. J. Invert. Path..

[2] Dively G.P., Kuhar T.P., Taylor S.V., Doughty H., Holmstrom K., Gilrein D.O., Nault B.A., Ingerson-Mahar J., Huseth A., Reisig D. (2023). Extended sentinel monitoring of *Helicoverpa zea* resistance to Cry and Vip3Aa toxins in Bt sweet corn: Assessing changes in phenotypic and allele frequencies of resistance. Insects.

[3] (2019). Brief 55: Global Status of Commercialized Biotech/GM Crops.

[4] Fleming D., Musser F., Reisig D., Greene J., Taylor S., Parajulee M., Lorenz G., Catchot A., Gore J., Kerns D. (2018). Effects of transgenic *Bacillus thuringiensis* cotton on insecticide use, heliothine counts, plant damage, and cotton yield: A meta-analysis, 1996–2015. PLoS ONE.

[5] Bueno A.d.F., Braz-Zini E.C., Horikoshi R.J., Bernardi O., Andrade G., Sutil W.P. (2025). Over 10 years of Bt soybean in Brazil: Lessons, benefits, and challenges for its use in integrated pest management (IPM). Neotrop. Entomol..

[6] Wu F. (2022). Mycotoxin risks are lower in biotech corn. Curr. Opin. Biotechnol..

[7] Lee M.K., Walters F.S., Hart H., Palekar N., Chen J.S. (2003). The mode of action of the *Bacillus thuringiensis* vegetative insecticidal protein Vip3A differs from that of Cry1Ab delta-endotoxin. Appl. Environ. Microbiol..

[8] Gilreath R.T., Kerns D.L., Huang F., Yang F. (2021). No positive cross-resistance to Cry1 and Cry2 proteins favors pyramiding strategy for management of Vip3Aa resistance in *Spodoptera frugiperda*. Pest Manag. Sci..

[9] Tabashnik B.E., Carriere Y. (2017). Surge in insect resistance to transgenic crops and prospects for sustainability. Nat. Biotechnol..

[10] Reisig D., Buntin G.D., Greene J.K., Paula-Moraes S.V., Reay-Jones F., Roberts P., Smith R., Taylor S.V. (2023). Magnitude and extent of *Helicoverpa zea* resistance levels to Cry1Ac and Cry2Ab2 across the Southeastern USA. Insects.

[11] Dively G.P., Venugopal P.D., Finkenbinder C. (2016). Field-evolved resistance in corn earworm to Cry proteins expressed by transgenic sweet corn. PLoS ONE.

[12] Gassmann A.J., Reisig D.D. (2023). Management of insect pests with Bt crops in the United States. Annu. Rev. Entomol..

[13] Yang F., Kerns D.L., Little N.S., Santiago González J.C., Tabashnik B.E. (2021). Early warning of resistance to Bt toxin Vip3Aa in *Helicoverpa zea*. Toxins.

[14] Gottems L. IMA Reveals Increased Insecticide Use in Viptera Biotechnology. AgNews 2025. https://news.agropages.com/News/NewsDetail---53498.htm.

[15] Ferré J., Bel Y., Lázaro-Berenguer M., Hernández-Martínez P., Jurat-Fuentes J.L. (2023). Chapter Three—Vip3 insecticidal proteins: Structure and mode of action. Advances in Insect Physiology.

[16] Lee M.K., Miles P., Chen J.S. (2006). Brush border membrane binding properties of *Bacillus thuringiensis* Vip3A toxin to *Heliothis virescens* and *Helicoverpa zea* midguts. Biochem. Biophys. Res. Commun..

[17] Tabashnik B.E., Carrière Y. (2020). Evaluating cross-resistance between Vip and Cry toxins of *Bacillus thuringiensis*. J. Econ. Entomol..

[18] Yu C.G., Mullins M.A., Warren G.W., Koziel M.G., Estruch J.J. (1997). The *Bacillus thuringiensis* vegetative insecticidal protein Vip3A lyses midgut epithelium cells of susceptible insects. Appl. Environ. Microbiol..

[19] Núñez-Ramírez R., Huesa J., Bel Y., Ferré J., Casino P., Arias-Palomo E. (2020). Molecular architecture and activation of the insecticidal protein Vip3Aa from *Bacillus thuringiensis*. Nat. Commun..

[20] Caccia S., Chakroun M., Vinokurov K., Ferré J. (2014). Proteolytic processing of *Bacillus thuringiensis* Vip3A proteins by two *Spodoptera* species. J. Insect Physiol..

[21] Zhang J., Pan Z.Z., Xu L., Liu B., Chen Z., Li J., Niu L.Y., Zhu Y.J., Chen Q.X. (2018). Proteolytic activation of *Bacillus thuringiensis* Vip3Aa protein by *Spodoptera exigua* midgut protease. Int. J. Biol. Macromol..

[22] Chakroun M., Bel Y., Caccia S., Abdelkefi-Mesrati L., Escriche B., Ferré J. (2012). Susceptibility of *Spodoptera frugiperda* and *S. exigua* to *Bacillus thuringiensis* Vip3Aa insecticidal protein. J. Invert. Pathol..

[23] Jiang K., Mei S.-q., Wang T.-t., Pan J.-h., Chen Y.-h., Cai J. (2016). Vip3Aa induces apoptosis in cultured *Spodoptera frugiperda* (Sf9) cells. Toxicon.

[24] Jiang K., Hou X.-Y., Tan T.-T., Cao Z.-L., Mei S.-Q., Yan B., Chang J., Han L., Zhao D., Cai J. (2018). Scavenger receptor-C acts as a receptor for *Bacillus thuringiensis* vegetative insecticidal protein Vip3Aa and mediates the internalization of Vip3Aa via endocytosis. PLoS Pathog..

[25] Hou X., Han L., An B., Zhang Y., Cao Z., Zhan Y., Cai X., Yan B., Cai J. (2020). Mitochondria and lysosomes participate in Vip3Aa-induced *Spodoptera frugiperda* Sf9 cell apoptosis. Toxins.

[26] Nimsanor S., Srisaisup M., Jammor P., Promdonkoy B., Boonserm P. (2020). Intracellular localization and cytotoxicity of *Bacillus thuringiensis* Vip3Aa against *Spodoptera frugiperda* (Sf9) cells. J. Invert. Pathol.

[27] Lázaro-Berenguer M., Ferré J., Hernández-Martínez P. (2024). Receptor interactions of protoxin and activated Vip3Aa structural conformations in *Spodoptera exigua*. Pest Manag. Sci..

[28] Barkhade U., Thakare A. (2010). Protease mediated resistance mechanism to Cry1C and Vip3A in *Spodoptera litura*. Egypt. Acad. J. Biol. Sci. A Entomol..

[29] Chakroun M., Banyuls N., Walsh T., Downes S., James B., Ferre J. (2016). Characterization of the resistance to Vip3Aa in *Helicoverpa armigera* from Australia and the role of midgut processing and receptor binding. Sci. Rep..

[30] Ayra-Pardo C., Ochagavía M.E., Raymond B., Gulzar A., Rodríguez-Cabrera L., Rodríguez de la Noval C., Morán Bertot I., Terauchi R., Yoshida K., Matsumura H. (2019). HT-SuperSAGE of the gut tissue of a Vip3Aa-resistant *Heliothis virescens* (Lepidoptera: Noctuidae) strain provides insights into the basis of resistance. Insect Sci..

[31] Roy R., Abdelgaffar H., Kerns D., Huff M., Staton M., Yang F., Huang F., Jurat-Fuentes J.L. (2025). Reduced processing and toxin binding associated with resistance to Vip3Aa in a strain of fall armyworm *(Spodoptera frugiperda)* from Louisiana. Pest Manag. Sci..

[32] Infante O., Gómez I., Pélaez-Aguilar A.E., Verduzco-Rosas L.A., García-Suárez R., García-Gómez B.I., Wang Z., Zhang J., Guerrero A., Bravo A. (2024). Insights into the structural changes that trigger receptor binding upon proteolytic activation of *Bacillus thuringiensis* Vip3Aa insecticidal protein. PLoS Pathog..

[33] Lázaro-Berenguer M., Paredes-Martínez F., Bel Y., Núñez-Ramírez R., Arias-Palomo E., Casino P., Ferré J. (2022). Structural and functional role of Domain I for the insecticidal activity of the Vip3Aa protein from *Bacillus thuringiensis*. Microb. Biotechnol..

[34] Jiang K., Chen Z., Shi Y., Zang Y., Shang C., Huang X., Zang J., Bai Z., Jiao X., Cai J. (2023). A strategy to enhance the insecticidal potency of Vip3Aa by introducing additional cleavage sites to increase its proteolytic activation efficiency. Eng. Microbiol..

[35] Qi L., Dai H., Jin Z., Shen H., Guan F., Yang Y., Tabashnik B.E., Wu Y. (2021). Evaluating cross-resistance to Cry and Vip toxins in four strains of *Helicoverpa armigera* with different genetic mechanisms of resistance to Bt toxin Cry1Ac. Front. Microbiol..

[36] Wang M., Zhang S., Shi Y., Yang Y., Wu Y. (2020). Global gene expression changes induced by knockout of a protease gene cluster in *Helicoverpa armigera* with CRISPR/Cas9. J. Insect Physiol..

[37] Sharath Chandra G., Asokan R., Manamohan M., Ellango R., Sharma H.C., Akbar S.M.D., Krishna Kumar N.K. (2018). Double-stranded RNA-mediated suppression of trypsin-like serine protease (t-SP) triggers over-expression of another t-SP isoform in *Helicoverpa armigera*. Appl. Biochem. Biotechnol..

[38] Jiang K., Chen Z., Zang Y., Shi Y., Shang C., Jiao X., Cai J., Gao X. (2023). Functional characterization of Vip3Aa from *Bacillus thuringiensis* reveals the contributions of specific domains to its insecticidal activity. J. Biol. Chem..

[39] Liu Z., Liao C., Zou L., Jin M., Shan Y., Quan Y., Yao H., Zhang L., Wang P., Liu Z. (2024). Retrotransposon-mediated variation of a chitin synthase gene confers insect resistance to *Bacillus thuringiensis* Vip3Aa toxin. PLoS Biol..

[40] Jin M., Shan Y., Peng Y., Chen S., Zhou X., Liu K., Xiao Y. (2024). CRISPR-mediated chromosome deletion facilitates genetic mapping of Vip3Aa resistance gene within complex genomic region in an invasive global pest. bioRxiv.

[41] Wang P., Liu Z., Kang Q., Liao C., Zou L., Mao K., Yao H., Li Y., Xiao Y. (2025). Functional loss of CHS2 confers high levels resistance to *Bacillus thuringiensis* Vip3Aa, but with significant fitness costs in five lepidopteran species. bioRxiv.

[42] Liu Z., Liao C., Zou L., Jin M., Shan Y., Quan Y., Yao H., Zhang L., Wang P., Liu Z. (2024). Retrotransposon-mediated variation of a chitin synthase gene confers insect resistance to *Bacillus thuringiensis* Vip3Aa toxin. bioRxiv.

[43] Chen X., Head G.P., Price P., Kerns D.L., Rice M.E., Huang F., Gilreath R.T., Yang F. (2019). Fitness costs of Vip3A resistance in *Spodoptera frugiperda* on different hosts. Pest Manag. Sci..

[44] Tandy P., Lamour K., Placidi de Bortoli C., Nagoshi R., Emrich S.J., Jurat-Fuentes J.L. (2023). Screening for resistance alleles to Cry1 proteins through targeted sequencing in the native and invasive range of *Spodoptera frugiperda* (Lepidoptera: Noctuidae). J. Econ. Entomol..

[45] Quan Y., Yang J., Wang Y., Hernández-Martínez P., Ferré J., He K. (2021). The Rapid Evolution of resistance to Vip3Aa insecticidal protein in *Mythimna separata* (Walker) is not related to altered binding to midgut receptors. Toxins.

[46] Abdelkefi-Mesrati L., Boukedi H., Chakroun M., Kamoun F., Azzouz H., Tounsi S., Rouis S., Jaoua S. (2011). Investigation of the steps involved in the difference of susceptibility of *Ephestia kuehniella* and *Spodoptera littoralis* to the *Bacillus thuringiensis* Vip3Aa16 toxin. J. Invert. Pathol..

[47] Chakrabarty S., Jin M., Wu C., Chakraborty P., Xiao Y. (2020). *Bacillus thuringiensis* vegetative insecticidal protein family Vip3A and mode of action against pest Lepidoptera. Pest Manag. Sci..

[48] Syed T., Askari M., Meng Z., Li Y., Abid M., Wei Y., Guo S., Liang C., Zhang R. (2020). Current insights on vegetative insecticidal proteins (Vip) as next generation pest killers. Toxins.

[49] Jurat-Fuentes J.L., Heckel D.G., Ferré J. (2021). Mechanisms of resistance to insecticidal proteins from *Bacillus thuringiensis*. Annu. Rev. Entom..

[50] Kerns D.D., Yang F., Kerns D.L., Stewart S.D., Jurat-Fuentes J.L. (2023). Reduced toxin binding associated with resistance to Vip3Aa in the corn earworm (*Helicoverpa zea*). Appl. Environ. Microbiol..

[51] Singh G., Sachdev B., Sharma N., Seth R., Bhatnagar R.K. (2010). Interaction of *Bacillus thuringiensis* vegetative insecticidal protein with ribosomal S2 protein triggers larvicidal activity in *Spodoptera frugiperda*. Appl. Environ. Microbiol..

[52] An B., Zhang Y., Li X., Hou X., Yan B., Cai J. (2022). PHB2 affects the virulence of Vip3Aa to Sf9 cells through internalization and mitochondrial stability. Virulence.

[53] Jiang K., Hou X., Han L., Tan T., Cao Z., Cai J. (2018). Fibroblast growth factor receptor, a novel receptor for vegetative insecticidal protein Vip3Aa. Toxins.

[54] Ben Hamadou-Charfi D., Boukedi H., Abdelkefi-Mesrati L., Tounsi S., Jaoua S. (2013). *Agrotis segetum* midgut putative receptor of *Bacillus thuringiensis* vegetative insecticidal protein Vip3Aa16 differs from that of Cry1Ac toxin. J. Invert. Pathol..

[55] Osman G.H., Altaf W.J., Saleh I.A.S., Soltane R., Abulreesh H.H., Arif I.A., Ramadan A.M., Osman Y.A. (2018). First report of detection of the putative receptor of *Bacillus thuringiensis* toxin Vip3Aa from black cutworm (*Agrotis ipsilon*). Saudi J. Biol. Sci..

[56] Shan Y., Jin M., Chakrabarty S., Yang B., Li Q., Cheng Y., Zhang L., Xiao Y. (2022). Sf-FGFR and Sf-SR-C are not the receptors for Vip3Aa to exert insecticidal toxicity in *Spodoptera frugiperda*. Insects.

[57] Liu J.-G., Yang A.-Z., Shen X.-H., Hua B.-G., Shi G.-L. (2011). Specific binding of activated Vip3Aa10 to *Helicoverpa armigera* brush border membrane vesicles results in pore formation. J. Invert. Pathol..

[58] Kunthic T., Watanabe H., Kawano R., Tanaka Y., Promdonkoy B., Yao M., Boonserm P. (2017). pH regulates pore formation of a protease activated Vip3Aa from *Bacillus thuringiensis*. Biochim. Et Biophys. Acta-Biomembr..

[59] Pacheco S., Gómez I., Peláez-Aguilar A.E., Verduzco-Rosas L.A., García-Suárez R., Do Nascimento N.A., Rivera-Nájera L.Y., Cantón P.E., Soberón M., Bravo A. (2023). Structural changes upon membrane insertion of the insecticidal pore-forming toxins produced by *Bacillus thuringiensis*. Front. Insect Sci..

[60] Hernández-Martínez P., Gomis-Cebolla J., Ferré J., Escriche B. (2017). Changes in gene expression and apoptotic response in *Spodoptera exigua* larvae exposed to sublethal concentrations of Vip3 insecticidal proteins. Sci. Rep..

[61] Tanaka S., Yoshizawa Y., Sato R. (2012). Response of midgut epithelial cells to Cry1Aa is toxin-dependent and depends on the interplay between toxic action and the host apoptotic response. FEBS J..

[62] Jurat-Fuentes J.L., Adang M.J. (2006). Cry toxin mode of action in susceptible and resistant *Heliothis virescens* larvae. J. Invert. Pathol..

[63] Bachler A., Padovan A., Anderson C.J., Wei Y., Wu Y., Pearce S., Downes S., James B., Tessnow A.E., Sword G.A. (2025). Disruption of HaVipR1 confers Vip3Aa resistance in the moth crop pest *Helicoverpa armigera*. PLoS Biol..

[64] Zhang Z., Wang L., Pang X., Tay W.T., Gordon K.H.J., Walsh T.K., Yang Y., Wu Y. (2024). Knockout of the *SfVipR1* gene confers high-level resistance to *Bacillus thuringiensis* Vip3Aa toxin in *Spodoptera frugiperda*. bioRxiv.

[65] Bachler A., Padovan A., Anderson C.J., Wei Y., Wu Y., Pearce S., Downes S., James B., Tessnow A., Sword G.A. (2024). Identification of a novel resistance gene which provides insight into Vip3Aa mode of action in *Helicoverpa armigera*. bioRxiv.

[66] Pinos D., Chakroun M., Millán-Leiva A., Jurat-Fuentes J.L., Wright D.J., Hernández-Martínez P., Ferré J. (2020). Reduced membrane-bound alkaline phosphatase does not affect binding of Vip3Aa in a *Heliothis virescens* resistant colony. Toxins.

[67] Jakka S.R.K., Knight V.R., Jurat-Fuentes J.L. (2014). *Spodoptera frugiperda* (J.E. Smith) with field-evolved resistance to Bt maize are susceptible to Bt pesticides. J. Invert. Pathol..

[68] Shabbir M.Z., Yang X., Batool R., Yin F., Kendra P.E., Li Z.-Y. (2021). *Bacillus thuringiensis* and chlorantraniliprole trigger the expression of detoxification-related genes in the larval midgut of *Plutella xylostella*. Front. Physiol..

[69] Wu Q.-L., He L.-M., Shen X.-J., Jiang Y.-Y., Liu J., Hu G., Wu K.-M. (2019). Estimation of the potential infestation area of newly-invaded fall armyworm *Spodoptera frugiperda* in the Yangtze River Valley of China. Insects.

[70] Jakka Siva R.K., Gong L., Hasler J., Banerjee R., Sheets Joel J., Narva K., Blanco Carlos A., Jurat-Fuentes Juan L. (2016). Field-evolved mode 1 resistance of the fall armyworm to transgenic Cry1Fa-expressing corn associated with reduced Cry1Fa toxin binding and midgut alkaline phosphatase expression. Appl. Environ. Microbiol..

[71] Banerjee R., Hasler J., Meagher R., Nagoshi R., Hietala L., Huang F., Narva K., Jurat-Fuentes J.L. (2017). Mechanism and DNA-based detection of field-evolved resistance to transgenic Bt corn in fall armyworm (*Spodoptera frugiperda*). Sci. Rep..

[72] Jin M., Shan Y., Peng Y., Wang W., Zhang H., Liu K., Heckel D.G., Wu K., Tabashnik B.E., Xiao Y. (2023). Downregulation of a transcription factor associated with resistance to Bt toxin Vip3Aa in the invasive fall armyworm. Proc. Natl. Acad. Sci. USA.

[73] Mahon R.J., Downes S.J., James B. (2012). Vip3A resistance alleles exist at high levels in Australian targets before release of cotton expressing this toxin. PLoS ONE.

[74] Bernardi O., Bernardi D., Ribeiro R.S., Okuma D.M., Salmeron E., Fatoretto J., Medeiros F.C.L., Burd T., Omoto C. (2015). Frequency of resistance to Vip3Aa20 toxin from Bacillus thuringiensis in *Spodoptera frugiperda* (Lepidoptera: Noctuidae) populations in Brazil. Crop Prot..

[75] Amaral F.S.A., Guidolin A.S., Salmeron E., Kanno R.H., Padovez F.E.O., Fatoretto J.C., Omoto C. (2020). Geographical distribution of Vip3Aa20 resistance allele frequencies in *Spodoptera frugiperda* (Lepidoptera: Noctuidae) populations in Brazil. Pest Manag. Sci..

[76] Yang F., Wang Z., Kerns D.L. (2022). Resistance of *Spodoptera frugiperda* to Cry1, Cry2, and Vip3Aa proteins in Bt corn and cotton in the Americas: Implications for the rest of the world. J. Econ. Entomol..

[77] Santiago-González J.C., Kerns D.L., Yang F. (2023). Resistance allele frequency of *Helicoverpa zea* to Vip3Aa *Bacillus thuringiensis* protein in the Southeastern U.S. Insects.

[78] Wen Z., Conville J., Matthews P., Hootman T., Himes J., Wong S., Huang F., Ni X., Chen J.S., Bramlett M. (2023). More than 10 years after commercialization, Vip3A-expressing MIR162 remains highly efficacious in controlling major Lepidopteran maize pests: Laboratory resistance selection versus field reality. Pestic. Biochem. Physiol..

[79] Carrière Y., Degain B., Unnithan G.C., Tabashnik B.E. (2023). Inheritance and fitness cost of laboratory-selected resistance to Vip3Aa in *Helicoverpa zea* (*Lepidoptera: Noctuidae*). J. Econ. Entomol..

[80] Bernardi O., Bernardi D., Horikoshi R.J., Okuma D.M., Miraldo L.L., Fatoretto J., Medeiros F.C., Burd T., Omoto C. (2016). Selection and characterization of resistance to the Vip3Aa20 protein from *Bacillus thuringiensis* in *Spodoptera frugiperda*. Pest Manag. Sci..

[81] Pickett Brian R., Gulzar A., Ferré J., Wright D.J. (2017). *Bacillus thuringiensis* Vip3Aa toxin resistance in *Heliothis virescens* (Lepidoptera: Noctuidae). Appl. Environ. Microbiol..

[82] Wang Y., Yang J., Zhang T., Bai S., Wang Z., He K. (2022). Inheritance and fitness costs of Vip3Aa19 resistance in *Mythimna separata*. Toxins.

[83] Yang F., Williams J., Huang F., Kerns D.L. (2021). Genetic basis and cross-resistance of Vip3Aa resistance in *Spodoptera frugiperda* (lepidoptera: Noctuidae) derived from Texas, USA. Crop Prot..

[84] Yang F., Head G.P., Kerns D.D., Jurat-Fuentes J.L., Santiago-González J.C., Kerns D.L. (2024). Diverse genetic basis of Vip3Aa resistance in five independent field-derived strains of *Helicoverpa zea* in the US. Pest Manag. Sci..

[85] Fabrick J.A., Wu Y., Jurat-Fuentes J.L. (2023). Chapter Four—Mechanisms and molecular genetics of insect resistance to insecticidal proteins from *Bacillus thuringiensis*. Advances in Insect Physiology.

[86] Gulzar A., Pickett B., Sayyed A.H., Wright D.J. (2012). Effect of temperature on the fitness of a Vip3A resistant population of *Heliothis virescens* (Lepidoptera: Noctuidae). J. Econ. Entomol..

[87] Yang F., Santiago González J.C., Sword G.A., Kerns D.L. (2021). Genetic basis of resistance to the Vip3Aa Bt protein in *Helicoverpa zea*. Pest Manag. Sci..

[88] Yang F., Morsello S., Head G.P., Sansone C., Huang F., Gilreath R.T., Kerns D.L. (2018). F2 screen, inheritance and cross-resistance of field-derived Vip3A resistance in *Spodoptera frugiperda* (Lepidoptera: Noctuidae) collected from Louisiana, USA. Pest Manag. Sci..

[89] Yang F., Williams J., Porter P., Huang F., Kerns D.L. (2019). F2 screen for resistance to *Bacillus thuringiensis* Vip3Aa51 protein in field populations of *Spodoptera frugiperda* (Lepidoptera: Noctuidae) from Texas, USA. Crop Prot..

[90] Tabashnik B.E., Fabrick J.A., Carrière Y. (2023). Global patterns of insect resistance to transgenic Bt crops: The first 25 years. J. Econ. Entomol..

[91] (2008). U.S. Environmental Protection Agency (EPA) Biopesticides Registration Action Document: *Bacillus thuringiensis* Modified Cry1Ab (SYN-IR67B-1) and Vip3Aa19 (SYN-IR102-7) Insecticidal Proteins and the Genetic Material Necessary for Their Production in COT102 X COT67B Cotton. https://www3.epa.gov/pesticides/chem_search/reg_actions/registration/decision_PC-006529_12-Aug-08.pdf.

[92] Chae H., Wen Z., Hootman T., Himes J., Duan Q., McMath J., Ditillo J., Sessler R., Conville J., Niu Y. (2022). eCry1Gb.1Ig, A novel chimeric Cry protein with high efficacy against multiple fall armyworm (*Spodoptera frugiperda*) strains resistant to different GM traits. Toxins.

[93] Wang Y., Wang J., Fu X., Nageotte Jeffrey R., Silverman J., Bretsnyder Eric C., Chen D., Rydel Timothy J., Bean Gregory J., Li K.S. (2019). *Bacillus thuringiensis* Cry1Da_7 and Cry1B.868 protein interactions with novel receptors allow control of resistant fall armyworms, *Spodoptera frugiperda* (J.E. Smith). Appl. Environ. Microbiol..

